# “She’s a Family Member”: How Community Health Workers Impact Perinatal Mothers’ Stress Through Social-Emotional Support and Connections to Programs and Resources

**DOI:** 10.1089/heq.2024.0038

**Published:** 2024-07-10

**Authors:** Justin Rex, Nichole Fifer, Karen D. Johnson-Webb, Maddi Menich, Alyissa Horn, Carly Salamone, Holly T. Renzhofer Pappada, Camelia Arsene, Crystal Martin, Malcolm Cunningham

**Affiliations:** ^1^ Political Science, Bowling Green State University, Bowling Green, Ohio, USA.; ^2^ Center for Regional Development, Bowling Green State University, Bowling Green, Ohio, USA.; ^3^ School of Earth, Environment, & Society, Bowling Green State University, Bowling Green, Ohio, USA.; ^4^ The Rucks Group, LLC, Dayton, Ohio, USA.; ^5^ Fisher-Titus Medical Center, Norwalk, Ohio, USA.; ^6^ Hospital Council of Northwest Ohio, Toledo, Ohio, USA.; ^7^ ProMedica Health System, University of Toledo School of Population Health, Toledo, Ohio, USA.; ^8^ Johns Hopkins Bloomberg School of Public Health, Baltimore, Maryland, USA.; ^9^ Mayor’s Office of Neighborhood Safety and Engagement, City of Toledo, Office of the Mayor, Toledo, Ohio, USA.

**Keywords:** health disparities, minority health, pregnancy, reproductive health

## Abstract

**Introduction:**

This study examines whether being a client in the Northwest Ohio Pathways HUB program reduces stress and improves mental wellbeing for perinatal mothers. The HUB works to improve health by connecting mothers to community health workers (CHWs) who assess mothers’ risk factors and connect them to evidence-based care pathways to reduce known risks associated with adverse birth outcomes.

**Methods:**

A one-time survey of 119 mothers in the program and monthly semi-structured interviews with 41 mothers, totaling 220 interviews.

**Results:**

Almost all mothers reported significantly reduced stress after joining the program. The majority also reported an improved sense of safety, security, and hope. Interviews show additional moderate reductions in stress over time while being a program client. Interviews also indicate that mothers’ relationship with their CHW is key to these improvements: CHW provide social-emotional support, access to tangible goods, and help navigating social service bureaucracies.

**Discussion:**

The results support the broader literature on the health benefits of community health workers and address identified gaps within the literature, which has infrequently studied CHWs in the perinatal context.

**Conclusion:**

CHWs may be one way to address racial inequity in birth outcomes linked to infant mortality, given research on the links between inequitable exposure to stressors, the impacts of racism-induced stress, and preterm and low birth weight babies. Further, the findings indicate the need to better support CHWs, and the programs that utilize them, with increased funding, insurance reimbursement, and certification.

## Introduction

Navigators, ranging from layperson community health workers (CHWs) to more professionalized personnel such as social workers and nurses, are liaisons and guides that help individuals navigate health care and community resources.^1^ For example, they help patients navigate complex and long-term medical conditions,^2^ provide patients social and emotional support,^3^ and help patients address the social determinants of health by assisting with issues related to housing, legal aid, employment, finances, and food insecurity.^4^ Numerous studies and systematic reviews indicate positive outcomes associated with navigators and CHWs, ranging from decreased emergency, hospital, and urgent care visits;^5^ increased screening and referrals;^6^ health improvements for chronic diseases;^7^ improved primary care and reduced days in the hospital;^8^ and reduced cardiovascular risk and more cost-effective care.^9^

Despite extensive research, scholars note that few studies have examined the benefits of these professionals from the perspective of patients themselves, instead focusing on clinical outcomes or the perspectives and opinions of health care professionals.^10^ Further, few studies examine navigators in the perinatal context,^11^ despite the fact that they commonly work in the area of pregnancy and prenatal care.^12–14^ Studies in this context have shown promising potential benefits, including increases in appointment attendance, contraceptive use, and vaccination rates,^15^ reduced preterm birth,^16^ and culturally informed care.^17^ This research parallels important findings from scholars that study patient perspectives on doula care and its positive impacts for patients and birth outcomes.^18,19^

Given these results, further research on navigators in the perinatal context is important, particularly given that they are intended to reduce health care disparities,^1^ which are well documented and stark for both mothers and children in the United States. For example, the largest inequities are between Black and non-Hispanic White women, with Black mothers’ pregnancy-related mortality rate nearly three times higher than that of White mothers and the Black infant mortality rate over twice as high as that of White infants.^20^ Reproductive justice scholarship connects these current inequities to a long history of reproductive injustice against birthing people of color embedded in our institutions, law, and policy, which still affect heath today and inform the movement’s human rights framework that prioritizes choice about whether to have a child, autonomy, and the right to parent in a safe and healthy environment.^21^

**FIG. 1.**
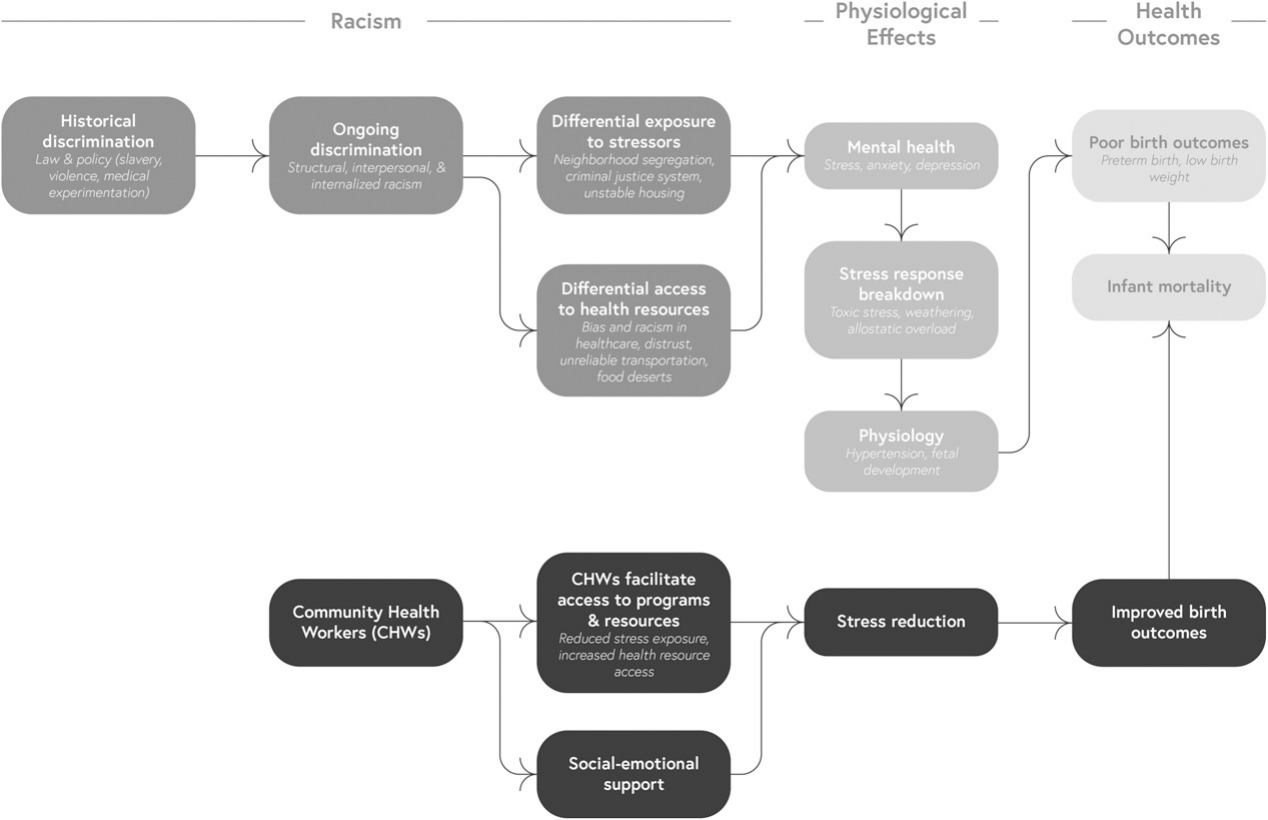
Theoretical Links Between Racism, Stress, and Birth Outcomes and the Impact of Community Health Workers (CHWs). This model is adapted from those developed by other scholars.^22–26^ It displays the theorized relationship between historical and ongoing racism, stress exposure, the impacts of stress on health during pregnancy, and birth outcomes. In addition, it shows the potential impact CHWs can have on stress by providing social-emotional support and improving access to programs and health resources.

A growing body of theoretical and empirical research argues that racism-induced stress is a key link between historical and current racism and birth outcomes.^22–26^ Theoretical models posit that historical discrimination embedded in law and policy creates current interpersonal, internalized, and structural racism, which cause racism-induced stress directly, but also increased exposure to other stressors; an overloaded stress response changes mothers’ physiology in ways that increase the likelihood of adverse birth outcomes.^22–26^ Empirical studies link stress-related racism from interpersonal discrimination, health care discrimination, neighborhood segregation, and interactions with the criminal justice and immigration enforcement systems, to disparities in low birth weight and/or preterm birth.^23^ Stressors can occur during pregnancy,^27,28^ preconception,^29^ or throughout one’s life course and have a cumulative effect on stress over time.^30–33^ Importantly, stress may result directly from instances of interpersonal racism in everyday life or in health care settings, but also from a legacy of structural racism that disproportionality exposes people color to health stressors while simultaneously disproportionately excluding them from easy access to the resources and institutions that could improve health.^34–36^ Given this evidence, scholars note the need to identify sources of resilience and potential mechanisms that may insulate mothers from the impact of racism-induced stress.^23,37^ CHWs could be one source of resilience, given the challenges they are trained to address (Fig. 1).

To better understand patient perspective about perinatal navigators and their potential to mitigate stressors resulting from structural racism, this study examines pregnant and recently pregnant mothers working with CHWs within the Northwest Ohio Pathways HUB. The program identifies potential clients, conducts a comprehensive risk assessment of 20 risk factors and social determinants of health known to be associated with poor birth outcomes (e.g., lack of insurance, food insecurity, unstable housing, unreliable transportation), and translates each risk factor into a care “pathway.” Clients are assigned CHWs employed at partnering government, medical, and social service care coordination agencies who assist mothers in completing pathways to reduce their identified health risks. The HUB establishes financial agreements with managed care organizations, public health authorities, and others, and the agencies employing CHWs are reimbursed for completed pathways. The Pathways Community HUB model is a nationally recognized, evidence-based best practice for improving birth outcomes and reducing health care spending.^38–40^ Overall, this program offers an avenue to examine the impacts of addressing inequitably distributed health resources and socio-economic stressors that are the product of structural racism and linked to racism-induced stress.

Using a survey and monthly semi-structured interviews with enrolled mothers over the course of up to 8 months during their program participation, we asked questions about sources of stress, changes over time to their stress and wellbeing, and service utilization to better understand the impacts of their CHWs. Interview and survey responses indicate that concerns about addressing social determinants of health, such as access to housing, health care, and food, are a frequent source of stress and that clients work with their CHWs to address these issues. Further, our data indicate program participation reduces stress and increases mothers’ sense of wellbeing. Lastly, interviews with mothers suggest CHWs are key to those improvements; CHWs provide social-emotional support, act as a liaison to tangible goods, and provide expertise to reduce the administrative burden associated with accessing public benefits. These findings are important for better understanding the impacts of CHWs and their ability to address perinatal health inequities.

### Methods

Interviewees were recruited using digital flyers distributed to clients via HUB staff, HUB partner organizations, and CHWs. Further, digital flyers were shared on local social media mom group pages and physical flyers were distributed to area food banks, agencies, and churches. CHWs were the primary method of recruitment given their previously established relationship with clients and the difficulty of reaching a population of mothers who frequently change phone numbers and addresses. To incentivize CHWs to assist with recruitment, we held two $100 gift card raffles in which each referral earned the CHW an entry into the drawing. Interviewees were compensated with a $50 mailed gift card for enrolling and an additional $50 mailed gift card for completing each interview. Grant funding for this project provided for a data collection period of 9 months, so mothers enrolled at the beginning of the data collection window had the opportunity to complete seven subsequent interviews, for a total of eight. Mothers who enrolled in the study later were enrolled using the same procedures but had fewer total interviews. During the interviews, mothers were asked a series of open-ended questions about their experience in the program. Some questions were the same for every interview, whereas others shifted as the mothers had more experience with the program. The data collection began after the COVID-19 shutdown, so interviews were conducted via phone call, video call, or using the free call features on social media sites for mothers who did not have phone data or a phone plan.

In total, the research team completed 220 interviews across 41 enrolled mothers who were referred by 11 different CHWs across 7 different partner agencies/organizations within the HUB network. Interviewees were asked an open-ended question about their race and almost half identified as Black or African American (46.3%), just under one-third as White (31.7%), about 12 percent multiracial (12.2%), and about 10% as Hispanic (9.8%). The racial demographics are similar to population demographics for all mothers in the program, with Black or African American (51.8%) and White (37.6%) mothers being slightly underrepresented in our sample, and multiracial (5%) and Hispanic, Latino/a, or Spanish Origin (0.6%) being overrepresented (Table 1).

**Table 1.** Race of Interviewees

Race	*n*	Percent
Black or African American	19	46.3%
White	13	31.7%
Multiracial	5	9.8%
Hispanic	4	12.2%
Total	41	100%

A flyer with a link to complete the survey was distributed to HUB clients in the same way as the interview flyer, with the addition that it was sent directly via text or email to mothers who were part of the interview sample. In total 119 mothers took the survey. Over half of survey respondents (58%) identified as Black or African American, whereas just under one-third identified as White (31.9%) and less than 10% as multiracial (6.7%) (Table 2). SPSS software was used to analyze survey results using a paired sample *t-test* and a one-way analysis of variance (ANOVA).

**Table 2.** Race of Survey Respondents

Race	*n*	Percent
Black or African American	69	58.0%
White	38	31.9%
Multiracial	8	6.7%
Other	4	3.4%
Total	119	100%

### Results

**FIG. 2.**
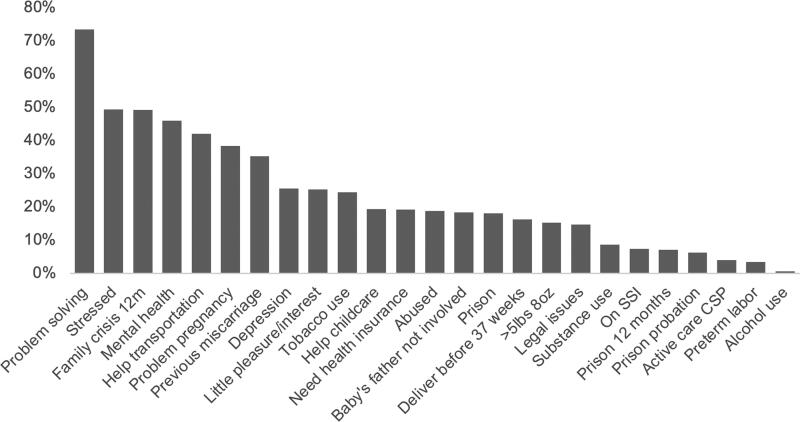
Risk Factors at Pathways HUB Enrollment. When clients enroll in the Pathways HUB, they complete an initial checklist with their CHW to screen them for known risk factors associated with infant mortality. Figure 1 shows the percentage of clients who indicated a risk factor at the time of their enrollment. The data are for mothers who enrolled between January 2018 and September 2020.

**FIG. 3.**
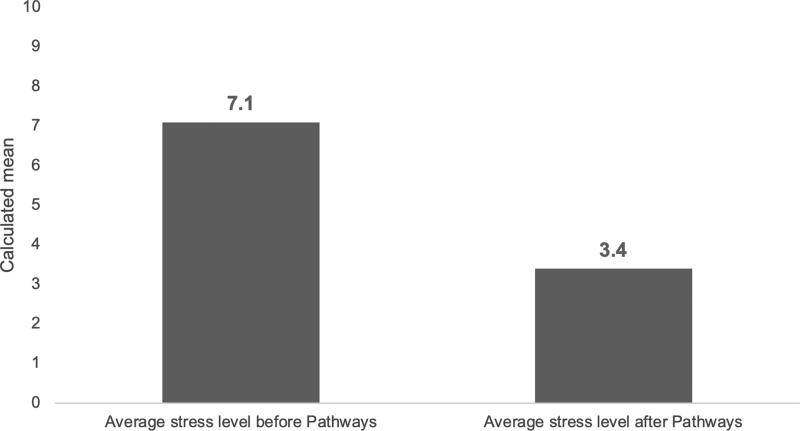
Mean Stress Level Before and After Pathways Enrollment. Clients were asked to rank their stress level before and after enrolling in the HUB: “On a scale of 1–10 (with 1 being the lowest amount of stress and 10 being the highest amount of stress), what would you say your stress level was before enrolling in the Pathways Hub Program?”; “On a scale of 1–10 (with 1 being the lowest amount of stress and 10 being the highest amount of stress), what would you say your average stress level was after enrolling in the Pathways Hub Program?”. Figure 2 shows that there was a substantively and statistically significant decrease in the self-reported stress level after enrolling. *Before: M = 7.12; SD = 2.66; After (M = 3.43; SD = 2.07); paired sample *t* test, t (114) = 14.08, *p* < 0.001). A one-way analysis of variance test (ANOVA) shows that there was not a statistically significant difference in the change in stress levels between the groups, F (3, 114) = 0.999, *p* = 0.39.

**FIG. 4.**
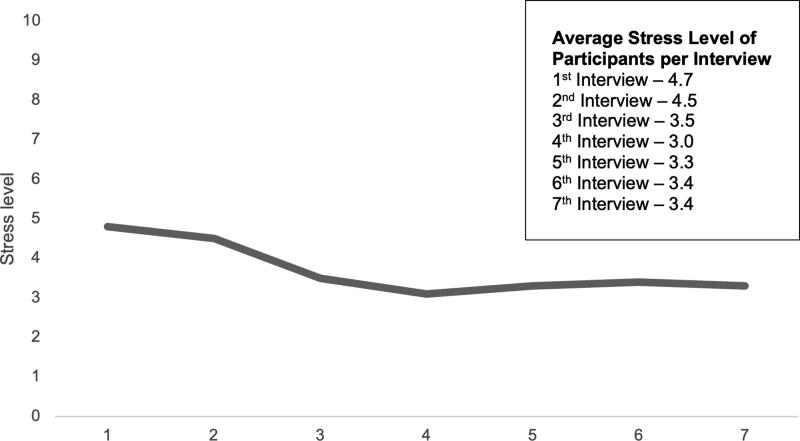
Average Stress Level at Time of Monthly Interview. Each month, interviewees were asked to indicate their stress level: First interview, “How is your current stress level on a scale of 1–10 (with 1 being low stress and 10 being high stress)?”; Subsequent interviews, “How is your current stress level? Last month when we spoke you said you were at a ___? Where would you rate your level now on a scale of 1 to 10 (with 1 being low stress and 10 being high stress?” This figure shows the mean stress level for mothers at each interview, indicating a small decrease in the average stress level over time.

When mothers enroll in the HUB, they complete a risk profile that asks about the occurrence of 20 known risk indicators associated with infant mortality. HUB program data indicating stress is the second most frequently identified risk factor, mentioned by slightly over half of clients at enrollment (Fig. 2). Survey respondents were asked a variety of questions to understand the effects participating in the HUB had on their mental wellbeing. First, mothers were asked to rank their stress level before and after enrolling in the HUB using a scale from 1 to 10, with 1 representing low stress and 10 indicating high stress levels. There was a substantively and statistically significant decrease in the mean reported stress after enrollment (Fig. 3), decreasing from 7.1 to 3.4, but there were not statistically or substantively significant differences in stress reduction between racial groups. One hundred and one respondents said their stress decreased, compared to eight who said they had no change in stress, and six who said their stress increased after enrollment. Interviewees were also asked to rank their stress on the same scale at each interview. Responses show that the mean stress level dropped slightly over their time in the program (Fig. 4).

**FIG. 5.**
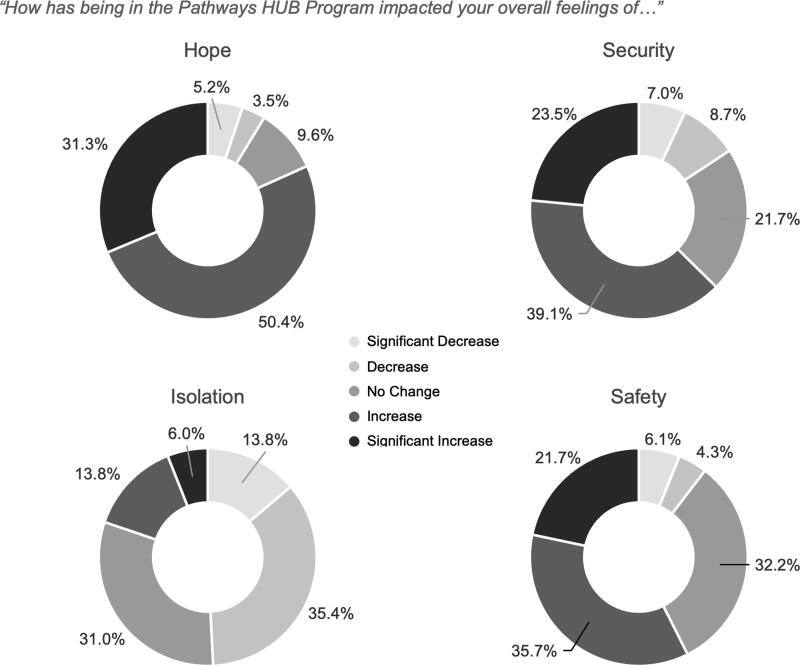
Effects of HUB Participation on Feelings of Hope, Security, Isolation, and Safety. Survey respondents were asked: 1) “How has being in the Pathways HUB program impacted your overall feelings of hope?” 2) “How has being in the Pathways HUB program impacted your overall feelings of security?” 3) “How has being in the Pathways HUB program impacted your overall feelings of safety?” 4) “How has being in the Pathways HUB program impacted your overall feelings of isolation?” For each question respondents were given the option to answer, “significantly decreased, decreased, no change, increased, or significantly increased.” Figure 4 indicates the percentage of respondents who chose a given option for each question.

Respondents were also asked about how being in the program impacted their overall feelings of security, safety, hope, and isolation. Most mothers said participation increased or significantly increased their overall feelings of security (60.5%) and safety (55.5%), and a large majority said participation increased their sense of hope (79%). Almost half of the respondents said participation decreased or significantly decreased their feelings of isolation (47.9%) (Fig. 5).

Additional data help us identify links between program participation and improvements in stress and wellbeing. During each interview, mothers were asked about what was currently causing their stress and worry. Often, they worried about risks to their health and economic wellbeing. For example, one pregnant mother worried about having insurance: “I got denied again [for Medicaid]. That’s what really raised my stress level. But [my CHW] said ‘Don’t even worry about it.’ She said she was looking at other things to help me with it. And that really calmed me down.” Another mom worried about finding housing and her CHW helped her apply for a housing voucher. “With [my CHW] it’s really easy because she always remembers the small little things. She makes paperwork a lot easier. When me and her do it together it feels like it goes ten times faster.” As one mother with persistent housing issues their landlord refused to address explained, their CHW told them “about escrow and about how to break the lease legally.” Our survey data show that CHWs connect clients to resources, programs, and public benefits to address the types of issues they worry about, following the intended design of the program, to identify health risks and guide clients through pathways to reduce those risks (Table 3). The most frequently mentioned supports were financial support programs such as food and cash assistance, followed by housing, insurance, and mental health services.

**Table 3.** CHW: Client Service Utilization

Service	Percent	*n*
Accessing social/financial support systems (WIC, Food Assistance, TANF, etc.)	69.6%	80
Housing Assistance	36.5%	42
Accessing medical insurance (Medicaid)	32.2%	37
Accessing mental health services	32.2%	37
Access to education and training opportunities	31.3%	36
Finding a doctor (primary care doctor, OBGYN, Pediatrician)	30.4%	35
Access to transportation	14.8%	17
Access to employment	13.9%	16
Access to childcare	12.2%	14
Other	14.8%	17

What services have you received through your participation with the Pathways HUB program working with your Community Health Worker? Please check all that apply.

WIC, Women, Infants, and Children; TANF, Temporary Assistance for Needy Families.

In addition, during the first interview, moms were asked directly what specific component of the program helped with their stress. Mothers consistently indicated that their CHW was a driver of reduced stress. Partly, this improvement was the strong bond that often developed between the mom and the CHW, and the social-emotional support CHWs provided. According to one mom, “I can talk to her about anything. I feel like she’s a family member.” Another mom said, “Honestly, [my CHW] as a person. If she’s at my doctor’s appointment or she comes to see me, it reduces a lot of stress if I mention something there and she calms me down…Her being there and knowing she’s at the office is really calming and stress reducing.” Further, CHWs were able to provide support by connecting moms to tangible goods and resources. “The little stuff she did was big for me” said one mom. According to another, “When I was about 7–8 months pregnant, I was nervous about having what I needed since I didn’t have a baby shower and she looked out for me, and I appreciate that.” For another mom, “She helped me find the house that I own now. She pointed me in the direction of Facebook Marketplace because realtors were a headache. It’s perfect for the kids and great location.”

Lastly, moms said CHWs were essential in helping them navigate bureaucracies to access public benefits. “She helped me get my WIC [Special Supplemental Nutrition Program for Women, Infants, and Children]. She actually called the office because I hadn’t heard from them for about three weeks. She called them, and then the next thing I know, they called me the next day, and I was able to get my card.” Said another mom, “Yeah, it’s helped [with stress] for sure. I send the stuff to her and she faxes it to them [Job and Family Services] and I think before when I dropped stuff off, they were like ‘this is just from a person,’ but when she drops it off, they’re like ‘oh shit this is from Pathways’ and they do it faster.” When asked in the final interview about the biggest benefit of participating in the program, the mothers reiterated these themes around receiving tangible goods, social-emotional support, and help navigating social service programs (Table 4).

**Table 4.** Biggest Benefit of Program

Benefit	*n*	Percent
Resource Provision (e.g., diapers, wipes, crib)	10	76.9%
Social-emotional Support	5	38.5%
Social Service Support	4	30.8%
General Unspecified Help	2	15.4%
Total	13	

### Discussion

Program data show that stress is one of the most frequently mentioned risk factors mothers indicate when enrolling in the Pathways HUB. Our survey data show that participating in the program positively impacts clients’ stress levels and mental wellbeing, most of whom report significantly reduced stress after joining the program and additional moderate reductions in stress over time. A significant majority of mothers indicate an improved sense of safety, security, and hope, and slightly less than half mothers say they experienced a decrease in feelings of isolation. Further, CHWs contribute to these impacts: our data show that mothers often worry about issues related to the social determinants of health and that CHWs help them address these issues. In the interviews, mothers attribute stress reduction to the social-emotional support CHWs provide, as well as their assistance accessing tangible goods and navigating bureaucracy to access public benefits.

The results of this study have important implications for research and practice. The findings support the broader literature on the positive impacts of navigators and CHWs.^5–9^ They add to this literature by showing these impacts extend to the perinatal context and do so by hearing about these impacts from clients themselves, rather than from health care professionals or medical outcome data, as in most previous studies.^10^ Similar to the literature on patient perspectives in doula care, these findings demonstrate that additional support for perinatal mothers can reduce stress, provide emotional support, and fill gaps in the medical and social support systems.^18,19^

Further, for the literature on racism, stress, and birth outcomes, and the need to find insulating mechanisms against racism-induced stress, the findings suggest that the HUB model, and the CHWs at its heart, help insulate mothers from stress that results in part from structural racism and inequitable access to a safe environment for having and raising children. Clients in the program are disproportionately black or African American, so this program is largely, but not exclusively, addressing the health care and social support needs of those who are most exposed to these inequities. Key social determinants of health and elements of a safe environment for mothers and children are what the model is set up to address. Our findings indicate that CHWs help mothers directly address these social determinants, and in doing so, reduce some stress associated with them in the process. Further, by pairing mothers with CHWs, who are often past clients in the program or, like doulas, are often drawn from the communities they serve,^19^ the model contains a form of collaborative resilience. Given that mothers frequently stay in the program over multiple pregnancies, these benefits extend over time.

This study has several limitations. First, the study examined the Pathways HUB model in one location. While the Pathways Community HUB Institute (PCHI) accredits programs to ensure implementation fidelity across sites, further study in different contexts is important to determine the generalizability of these results. In addition, with the onset of the COVID-19 pandemic and shutdown taking place between when this research was designed and funded, but before data collection took place, the results are bound by a particular time and historical circumstances. Whether the impacts might be larger or smaller outside the context of a global pandemic needs further study. Further, given the challenges of recruiting and remaining in contact with a population that frequently changes addresses and phone numbers, there was some study attrition and late recruitment into the interview sample, which led to some participants not completing the full set of interviews. Lastly, literature on the links between discrimination, racism-induced stress, and birth outcomes indicates the need for further research and evidence to support this complex causal chain more robustly; although our research can inform this literature and suggest a potential way to mitigate stress, it speaks only to a part of this process, upstream from the harmful biological effects of stress and associated birth outcomes.^22^ As others note, there is a need for longitudinal research that tracks each of these variables over time to draw more conclusive connections.^22^

### Health equity implications

Our results showing CHWs contribute to reduced stress for mothers that results from inequitable exposure to socioeconomic stressors and inequitable access to health care resources, indicate the benefit of supporting CHWs through additional financial support and/or expanded adoption of the Pathways Community HUB model. The CHW profession faces several challenges, including high turnover, pay disparities, underfunding, short-term and unstable funding models, among others.^41,42^ To better support the profession, scholars recommend states adopt Medicaid reimbursement for CHWs in states without it, or higher reimbursement for those that already do.^41^ Further, expanding CHW certification is associated with higher wages in states that certify.^41^ To better support the HUB model, states should ensure that CHW reimbursement is based on payment for outcomes, not just fee for service. Ultimately, the findings here support the idea that CHWs, and the policies and programs that support them, should be part of the solution for ensuring mothers live in a safe and healthy environment to have and raise their children.

## Notes

### Abbreviations Used

CHW: Community Health Worker
HUB: Northwest Ohio Pathways HUB
